# Secure Virtual Network Provisioning over Key Programmable Optical Networks

**DOI:** 10.3390/e27101042

**Published:** 2025-10-07

**Authors:** Xiaoyu Wang, Hao Jiang, Jianwei Li, Zhonghua Liang

**Affiliations:** China Academy of Information and Communications Technology, Beijing 100191, China

**Keywords:** information-theoretic security, secure virtual networks, key programmable optical networks, software-defined networking

## Abstract

Virtual networks have emerged as a promising solution for enabling diverse users to efficiently share bandwidth resources over optical network infrastructures. Despite the invention of various schemes aimed at ensuring secure isolation among virtual networks, the security of data transfer in virtual networks remains a challenging problem. To address this challenge, the concept of evolving traditional optical networks into key programmable optical networks (KPONs) has been proposed. Inspired by this, this paper delves into the establishment of secure virtual networks over KPONs, in which the information-theoretically secure keys can be supplied for ensuring the information-theoretic security of data transfer within virtual networks. A layered architecture for secure virtual network provisioning over KPONs is proposed, which leverages software-defined networking to realize the programmable control of optical-layer resources. With this architecture, a heuristic algorithm, i.e., the key adaptation-based secure virtual network provisioning (KA-SVNP) algorithm, is designed to dynamically allocate key resources based on the adaption between the key supply and key demand. To evaluate the proposed solutions, an emulation testbed is established, achieving millisecond latencies for secure virtual network establishment and deletion. Moreover, numerical simulations indicate that the designed KA-SVNP algorithm performs superior to the benchmark algorithm in terms of the success probability of secure virtual network requests.

## 1. Introduction

The exponential growth of network traffic driven by Internet-based applications (e.g., cloud computing, augmented reality and e-banking) has led to an increased focus on the need for sensitive data secrecy. Optical networks have been widely deployed around the world and have become one of the most important underlying telecommunication facilities for data transfer nowadays. However, a variety of sophisticated and diverse attacks (e.g., eavesdropping, jamming and interception [1]) have emerged in optical networks, posing a significant threat to the optical network security.

Encryption is an effective method for diminishing the adverse effects of the aforementioned attacks and enhancing the security of data transmission through optical networks. It is exceedingly difficult for an eavesdropper to decrypt the ciphertext without knowledge of the encryption key. In order to ensure the adaptability and compatibility with high-speed optical networks, a variety of optical domain encryption strategies have been proposed [2,3,4,5,6,7], with the aim of improving the security of optical networks. Nevertheless, the enhanced computational capabilities and the invention of advanced algorithms [8,9] may compromise the security of numerous traditional secrecy techniques for optical networks in the near future.

The physical layer security (PLS) based methods have been proposed as effective means to augment the security of optical networks. One such method is the quantum noise stream cipher (QNSC) [10,11,12,13], which has been suggested as a method of masking the signal states with intrinsic quantum noise, thereby directly encrypting the data across optical networks. Furthermore, quantum key distribution (QKD) [14,15,16,17], in combination with optical networks, can provide complementary capacity and security, where the information-theoretically secure keys can be produced. The produced secure keys can be further combined with the one-time pad algorithm to guarantee the information-theoretic security of data encryption. Obviously, the secure keys are of paramount importance in providing enhanced security for future optical networks. In particular, the concept and architecture of key programmable optical networks (KPONs) have been proposed in [18], enabling the flexible generation and distribution of the secure keys using PLS-based approaches under the programmable techniques.

Currently, the virtual networks are being developed and constructed on top of the substrate optical network infrastructure with the objective of sharing valuable network resources (e.g., computing, switching and spectrum resources) [19,20,21,22]. This approach offers the advantage of improving the utilization of optical-layer resources and is, therefore, an essential component of the future optical network architecture. As the volume of confidential data transferred daily by the virtual networks continues to grow, the credibility of optical networks will become increasingly important. Hence, it is anticipated that the KPON will evolve to serve as the substrate optical network for the realization of secure virtual networks, in which the information-theoretically secure keys can be supplied for ensuring the information-theoretic security of data transfer within virtual networks. Accordingly, how to efficiently realize the provisioning of numerous secure virtual networks over a KPON becomes an essential problem.

Targeting at this problem, this work proposes a layered architecture for secure virtual network provisioning over KPONs, in which a software-defined networking (SDN) controller is deployed in the middle layer for efficient resource management, and the programmable agent is developed to enable the flexible configuration of KPONs. In addition, a key adaptation-based secure virtual network provisioning (KA-SVNP) algorithm is devised, where the information-theoretically secure key resources and the key requirements are specially taken into account in the construction of multiple secure virtual optical networks. Further, an emulation testbed is established, while the total latency for secure virtual network establishment and deletion, as well as the average control latency for a single node, are evaluated. The success probability of secure virtual network requests is evaluated through numerical simulations, demonstrating the superior performance of the devised KA-SVNP algorithm in comparison to the benchmark algorithm.

The rest of this paper is organized as follows. Section 2 presents the network architecture for secure virtual network provisioning over KPONs. The network model and the KA-SVNP algorithm are proposed in Section 3. Section 4 demonstrates the emulation results and analyzes the simulation results. Finally, Section 5 concludes this paper.

## 2. Network Architecture

### 2.1. Securing Virtual Networks with PLS-Based Keys

**Figure 1:**
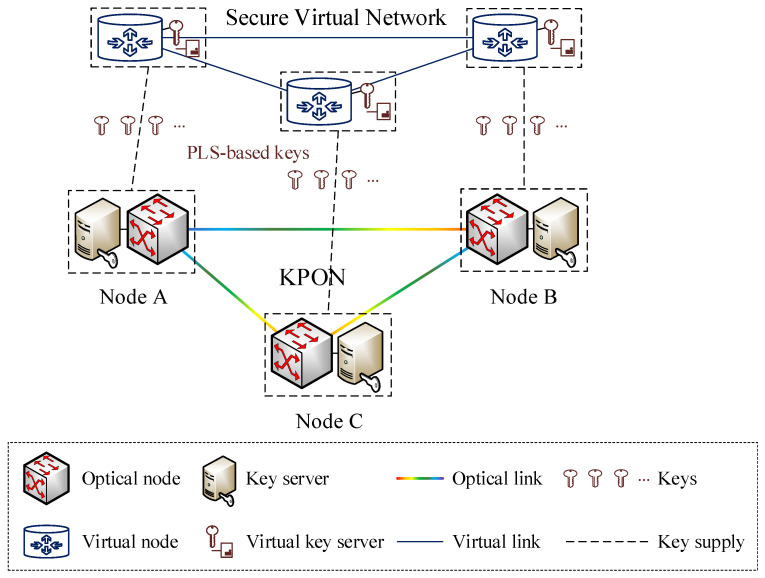
Illustration of securing virtual networks with PLS-based keys from the KPON.

The PLS techniques for key generation and distribution in optical networks allow for the production of information-theoretically secure keys between any pair of optical nodes. These optical nodes are situated within the substrate nodes of the KPON. Figure 1 illustrates the process of securing virtual networks with PLS-based keys from the KPON. The key server is situated in the same physical location as the optical node, in order to manage the entire lifetime of keys with information-theoretic security, including key generation, distribution, allocation, destruction, and so on. To elaborate a little further, the key generation and distribution are implemented based on the PLS module (e.g., QKD module) embedded within the key server. Furthermore, the specific implementation details of key management (e.g., key storage, allocation, updating, and destruction) are not the focus of this paper. Specifically, we adopt the standard key management operations specified in ITU-T Recommendation Y.3803 [23]. For further details of the operations of key management, please refer to [23]. Notably, the forward security is supported since the generated keys can ensure information-theoretic security based on PLS-based approaches (e.g., QKD). In light of the fact that PLS-based keys are generated in a distributed manner, it is necessary to place the key server in a distributed manner. This entails equipping each optical node with a key server. Multiple virtual networks can be constructed over the same KPON. Each virtual network is constituted of several virtual nodes interconnected by virtual links. The transfer of data across the virtual network can be secured by obtaining the requisite secure keys from the key server. The key server is tasked with realizing the virtualization of PLS-based key resources, thereby facilitating the allocation of secure keys for data encryption and decryption between each pair of virtual nodes. The virtual nodes and virtual links are abstracted from the optical nodes and optical links in the KPON, respectively. The key requirements for different pairs of virtual nodes may be different. Hence, the construction of a secure virtual network has to consider not only the bandwidth resource requirements of the virtual links, but also the key requirements between each pair of virtual nodes.

As exemplified in Figure 1, in the event that the information-theoretically secure keys shared between any pair of optical nodes (e.g., Nodes A and B) are symmetric keys, a proportion of these keys should be symmetrically virtualized into virtual key servers situated between virtual nodes A and B, in order to satisfy the key requirements of the secure virtual network. In particular, the security requirements of the virtual network are specific to each pair of virtual nodes. Moreover, the virtual link A-B is abstracted from the optical link A-B by occupying a portion of the bandwidth resources. In consequence of the aforementioned process, the virtual network is able to use bandwidth resources and information-theoretically secure key resources for secure communications among its virtual nodes, without concerning itself with the specifics of the underlying KPON infrastructure.

### 2.2. Layered Architecture for Secure Virtual Network Provisioning over KPONs

**Figure 2:**
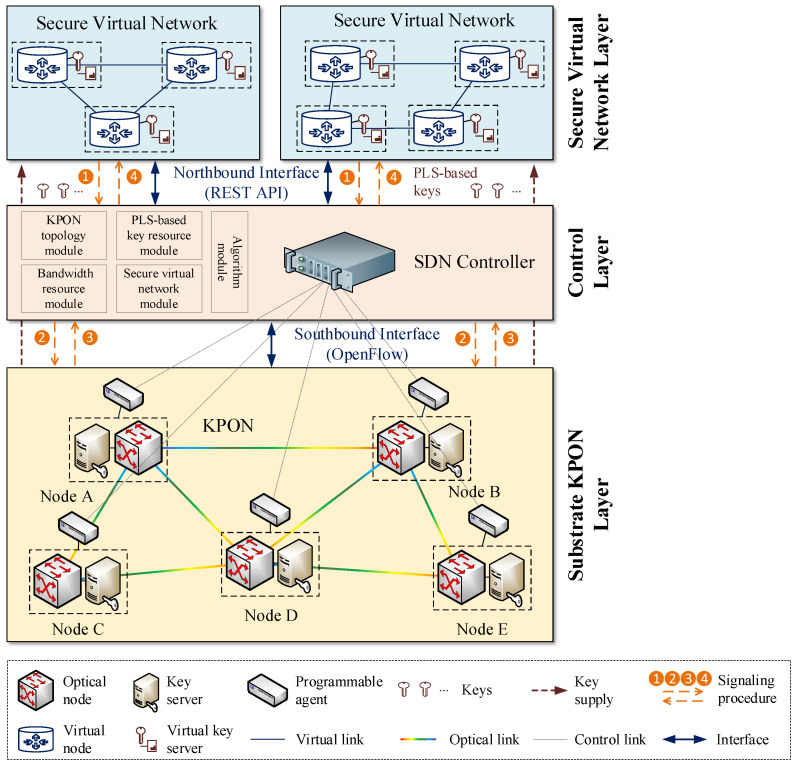
Illustration of the layered architecture for secure virtual network provisioning over KPONs.

In order to ensure the effective implementation of the aforementioned process for securing virtual networks with information-theoretically secure keys from the KPON, it is advisable to introduce the SDN technique, which allows for the centralized management of the KPON via a logical control approach. The feasibility of introducing the SDN technique with an efficient global control capability for virtual network construction has been confirmed in previous studies [24,25]. Accordingly, the implementation of secure virtual network provisioning over KPONs can rely on the SDN-based layered architecture shown in Figure 2. This layered architecture comprises three layers, arranged in a top-down sequence, i.e., the secure virtual network layer, the control layer and the substrate KPON layer. The substrate KPON layer is constituted of a number of substrate nodes interconnected by substrate fiber links. Each substrate node comprises an optical node and a key server. The keys with information-theoretic security are generated between each pair of optical nodes and their life cycles are managed by the corresponding key servers. In addition, each substrate node is linked to a programmable agent to facilitate its adaptive configuration.

The secure virtual network layer is constituted by a number of secure virtual networks, which are abstracted from the substrate KPON layer. A secure virtual network consists of several virtual nodes interconnected by virtual links, where the virtual key servers present in each virtual node are abstracted from the corresponding key servers. Specifically, a subset of keys with information-theoretic security is virtualized from the key server and stored in the virtual key server, in order to satisfy the corresponding security requirements of each virtual network. In reality, multiple secure virtual networks can share the bandwidth and key resources over the same KPON.

In the control layer, a centralized SDN controller is deployed with the objective of performing flexible secure virtual network provisioning over the KPON. The SDN controller comprises a number of modules, each of which implements a specific control function for the KPON. These include the KPON topology module, the bandwidth resource module, the PLS-based key resource module, the secure virtual network module, and the algorithm module. As the control layer represents a middle layer within this layered architecture, it also serves as a bridge between the secure virtual network layer and the substrate KPON layer. The northbound interface is employed for the intercommunication between the control layer and the secure virtual network layer, with the REST API serving as the implementation. Conversely, the southbound interface is implemented by OpenFlow [26,27], which facilitates communication between the control layer and the substrate KPON layer. The simplified control and configuration signaling procedures for secure virtual network provisioning are indicated by orange dashed lines and numbered from 1 to 4. Furthermore, the security of these control and configuration signaling messages can be augmented through the use of keys with information-theoretic security.

## 3. Heuristic Algorithm Design

### 3.1. Network Model

The network model entails the mathematical representation of the substrate KPON and the secure virtual network request. A KPON is modelled as $G^{s}\left(V^{s},E^{s}\right)$, where $V^{s}$ and $E^{s}$ represent the substrate node set and the substrate fiber link set, respectively. Additionally, a substrate node is denoted by $v\in V^{s}$. In accordance with the aforementioned description, the optical nodes and the key servers also correspond to $V^{s}$. On each substrate link $e\in E^{s}$, the total bandwidth resources and the total information-theoretically secure key resources are denoted by $B_{e}$ and $K_{e}$, respectively. It is important to note that the bandwidth resources are reflected by the number of wavelength channels, while the information-theoretically secure key resources are reflected by the PLS-based key rates.

The set of incoming secure virtual network requests is modelled as $G^{o}$. Each secure virtual network request is represented by $r\left(V_{r}^{o},E_{r}^{o}\right)$, where $V_{r}^{o}$ and $E_{r}^{o}$ represent the virtual node set and the virtual link set, respectively. In addition, a virtual node of request r is denoted by $v_{r}\in V_{r}^{o}$, while a virtual link of request r is denoted by $e_{r}\in E_{r}^{o}$. Furthermore, each secure virtual network request is associated with specific bandwidth requirements and key requirements for the implementation of secure communications among its virtual nodes. In this section, for each secure virtual network request, it is assumed that the bandwidth requirement for each virtual link is the same and the key requirement between each pair of virtual nodes is the same. The bandwidth requirements and key requirements of request r are represented by $b_{r}$ and $k_{r}$, respectively.

Given that the bandwidth resources and the information-theoretically secure key resources can be employed dynamically, the real-time available bandwidth resources and the information-theoretically secure key resources for link e at time step t are represented by $B_{e}^{t}$ and $K_{e}^{t}$, respectively. These can be expressed as follows:

(1) $$B_{e}^{t}=B_{e}-\sum_{r\in N_{e}^{t}}b_{r}$$

(2) $$K_{e}^{t}=K_{e}-\sum_{r\in N_{e}^{t}}k_{r}$$

where $N_{e}^{t}$ represents the set of online secure virtual networks successfully configured over the KPON that occupy link e at time step t.

In this paper, we focus on enhancing the security of virtual networks. Consequently, the information-theoretically secure key resources are the primary resources considered for the construction of secure virtual networks. For each virtual link, it may be abstracted from a physical path on the substrate KPON, given that the substrate nodes corresponding to the virtual nodes may not be directly connected. The set of physical paths available for virtual link $e_{r}$ is denoted by $P(e_{r})$, where each physical path is denoted by $p(e_{r})\in P(e_{r})$. Then, the set of substrate links on path $p(e_{r})$ is represented by $E_{p(e_{r})}$. The real-time available information-theoretically secure key resources for the physical path $p(e_{r})$ at time step t is denoted by $K_{p(e_{r})}^{t}$, which is characterized by the minimum available information-theoretically secure key resources on the substrate links along the path $p(e_{r})$. A key adaption metric is defined for the path $p(e_{r})$ as follows:

(3) $$D_{p(e_{r})}=\frac{K_{p(e_{r})}^{t}-k_{r}}{\left|E_{p(e_{r})}\right|}$$

In the event that the value of $D_{p(e_{r})}$ is negative, it becomes impossible to satisfy the key requirements of the corresponding secure virtual network request. In practice, the path with the maximum value of $D_{p(e_{r})}$ is the better option in $P(e_{r})$ for implementing the virtual link $e_{r}$ of this request, since the achievable information-theoretically secure key resources are more sufficient and the number of hops is lower in this case, thus contributing to the saving of information-theoretically secure key resources.

### 3.2. KA-SVNP Algorithm

A dynamic key adaptation-based secure virtual network provisioning (KA-SVNP) algorithm is devised to map each incoming secure virtual network request over the KPON, as shown in Algorithm 1. This can be expressed as the secure virtual network mapping for each request r, involving two primary steps: firstly, node mapping for each virtual node, i.e., $v_{r}$ can be mapped onto a set of candidate substrate nodes (i.e., $V^{s}$); secondly, link mapping for each virtual link, i.e., $e_{r}$ can be mapped onto a set of candidate physical paths (i.e., $P(e_{r})$). In this context, a deterministic relationship is established between the virtual node and the substrate node for a secure virtual network request. The virtual link $e_{r}$ is mapped into one of the alternative physical paths $p(e_{r})$, where the bandwidth resource constraint and the information-theoretically secure key resource constraint are considered. It is important to note that the allocation of key resources takes precedence over that of bandwidth resources, given the necessity for securing the virtual network prior to communication between its virtual nodes. The K-shortest-path approach is utilized for computing alternative physical paths for each node pair. It employs edge weights calculated based on hop counts and does not involve key resources. Subsequently, the final path is selected based on the adaption between the key supply and key demand (i.e., the path with the maximum value of $D_{p(e_{r})}$ is selected). The final assignment also depends on the ordering of virtual links, which is based on the number of hops in their corresponding final physical paths, arranged in ascending order. Furthermore, the first-fit approach is adopted for the allocation of bandwidth and information-theoretically secure key resources, which has been extensively utilized for resource allocation in secure optical networks [28,29]. The failure of secure virtual network requests typically stems from insufficient bandwidth or key resources, while the search strategy for resource allocation (e.g., the first-fit approach used in this paper) can also contribute to such failures. Hence, the search strategy for resource allocation can be further optimized in future studies. It is important to note that existing solutions (including the designed KA-SVNP algorithm) to the virtual network provisioning problem are difficult to achieve optimality in dynamic scenarios, which has been proven to be an NP-hard problem [21].
**Algorithm 1: KA-SVNP algorithm****Input:**  $G^{s}\left(V^{s},E^{s}\right)$, $B_{e}$, $K_{e}$, $G^{o}$, $b_{r}$, $k_{r}$**Output:** $M\left(r\right)$, $G^{w}$, SP1:  initialize $G^{w}\leftarrow \emptyset$;2:  **for** each incoming secure virtual network request $r\left(V_{r}^{o},E_{r}^{o}\right)$
**do**3:        **for** each virtual node $v_{r}\in V_{r}^{o}$
**do**4:           perform deterministic node mapping to map the virtual node from the secure             virtual network request onto a substrate node, where the assignment is            realized for the virtual node with the same location as the substrate node; 5:        **end for**6:        **for** each virtual link $e_{r}\in E_{r}^{o}$
**do**7:           compute the alternative physical paths with the K-shortest-path approach;8:           record the alternative physical paths in $P(e_{r})$;9:           **for** each alternative physical path $p(e_{r})\in P(e_{r})$
**do**10:               search the substrate links on path $p(e_{r})$and record them in $E_{p(e_{r})}$;11:               search the real-time available information-theoretically secure key                resources for each substrate link along the physical path $p(e_{r})$;12:               search the real-time available bandwidth resources for each substrate link                along the physical path $p(e_{r})$;13:               compute the real-time available information-theoretically secure key                resources $K_{p(e_{r})}^{t}$for the physical path $p(e_{r})$;14:               $D_{p(e_{r})}\leftarrow \frac{K_{p(e_{r})}^{t}-k_{r}}{\left|E_{p(e_{r})}\right|}$;15:               **if** $D_{p(e_{r})}<0$
**do**16:                   remove this physical path from $P(e_{r})$;17:               **end if**18:               **if** the bandwidth requirement cannot be satisfied **do**19:                   remove this physical path from $P(e_{r})$;20:               **end if**21:          **end for**22:          **if** $\left|P(e_{r})\right|\geq 1$do23:               mark X←SUCCEED;24:               select the alternative physical path with the maximum value of $D_{p(e_{r})}$ in                 $P(e_{r})$ as the final physical path for $e_{r}$;25:          **else**26:               mark X←FAILED;27:               **break**;28:          **end if**29:       **end for**30:       **if**  X=SUCCEED
**do**31:           record the secure virtual network request $r\left(V_{r}^{o},E_{r}^{o}\right)$ in $G^{w}$;32:           allocate the information-theoretically secure key resources and the            bandwidth resources with the first-fit approach;33:           update the status of information-theoretically secure key resources and            bandwidth resources;34:       **else**35:           the secure virtual network request is failed;36:       **end if**37:**end for**38:estimate the success probability: $SP\leftarrow \frac{\left|G^{w}\right|}{\left|G^{o}\right|}$;39:**return**  $M\left(r\right)$, $G^{w}$, SP.

The success probability (SP) of secure virtual network requests is estimated based on the ratio of the number of successfully provisioned secure virtual network requests to the total number of incoming secure virtual network requests, which can be expressed as:

(4) $$SP=\frac{\left|G^{w}\right|}{\left|G^{o}\right|}$$

where $G^{w}$ represents the set of secure virtual network requests that are successfully provisioned over the KPON.

## 4. Evaluation and Analysis

### 4.1. Emulation Results for the Latency

**Figure 3:**
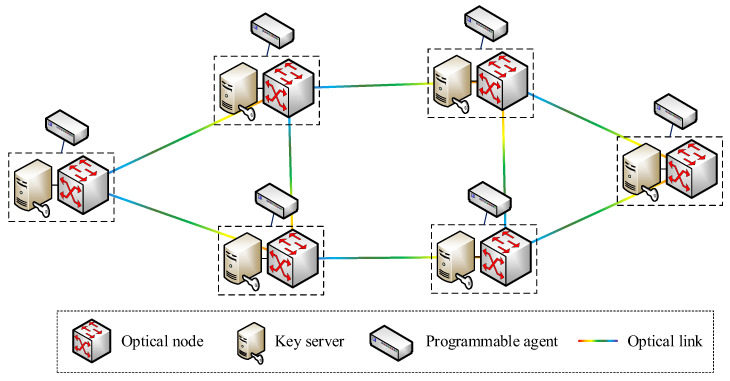
The KPON topology used in the emulation.

In order to verify the proposed layered architecture for secure virtual network provisioning over KPONs, an emulation testbed is established, following the KPON topology shown in Figure 3. The REST API is developed based on the HTTP/JSON, while the OpenFlow protocol is implemented for the intercommunication between the SDN controller and programmable agents. The SDN controller is developed based on the OpenDayLight platform. Moreover, the Open vSwitch is developed to implement the function of the programmable agent, which also emulates the functions of optical nodes and key servers.

**Figure 4:**
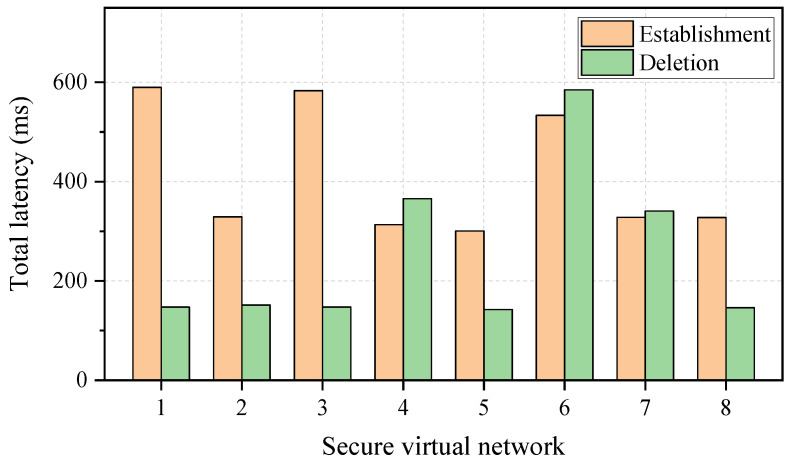
The total latency for the establishment/deletion of secure virtual networks.

Figure 4 displays the establishment and deletion latencies for secure virtual networks. The number of virtual nodes for each virtual network is set to 3, while the PLS-based key requirement and bandwidth requirement are set to 100 bps and 50 GHz (i.e., a wavelength channel), respectively. The total latency represents the time interval between the arrival of the secure virtual network establishment/deletion request and the reception of the establishment/deletion response. It can be observed that the total latencies exhibit variability across different secure virtual networks. In the case of the emulated eight requests, the minimum and maximum total latencies for the establishment of the secure virtual network are 300.5 ms and 589.9 ms, respectively. In addition, the minimum and maximum total latencies for the deletion of the secure virtual network are 142.6 ms and 584.8 ms, respectively. Hence, even though the total latency may fluctuate, the millisecond latency for the establishment/deletion of a secure virtual network over the KPON can be achieved.

**Figure 5:**
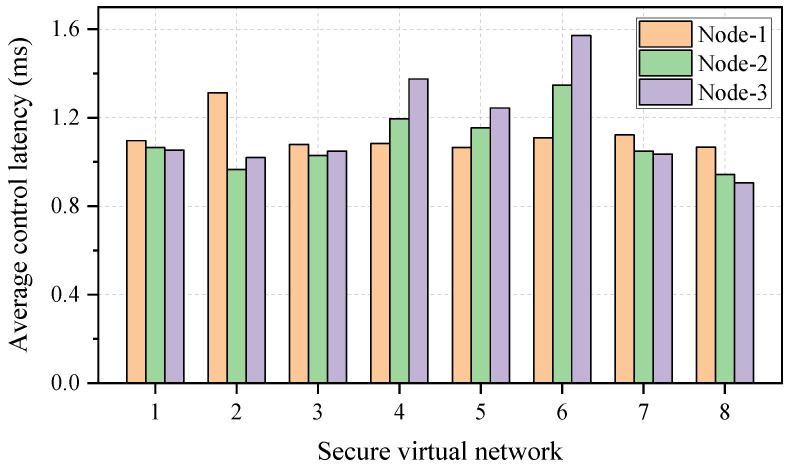
The average control latency for the intercommunication between the SDN controller and a single node.

The emulation results of the average control latency for the intercommunication between the SDN controller and a single node are illustrated in Figure 5. It can be observed that the average control latency is lower than 2 ms for a once-only intercommunication, demonstrating the benefits of the KPON in supporting the low-latency configuration of secure virtual networks. In addition, the virtual nodes/links for a secure virtual network are processed individually in the emulation testbed, hence the latency will increase in proportion to the scale of the virtual network.

### 4.2. Simulation Results for the Success Probability of Secure Virtual Network Requests

**Figure 6:**
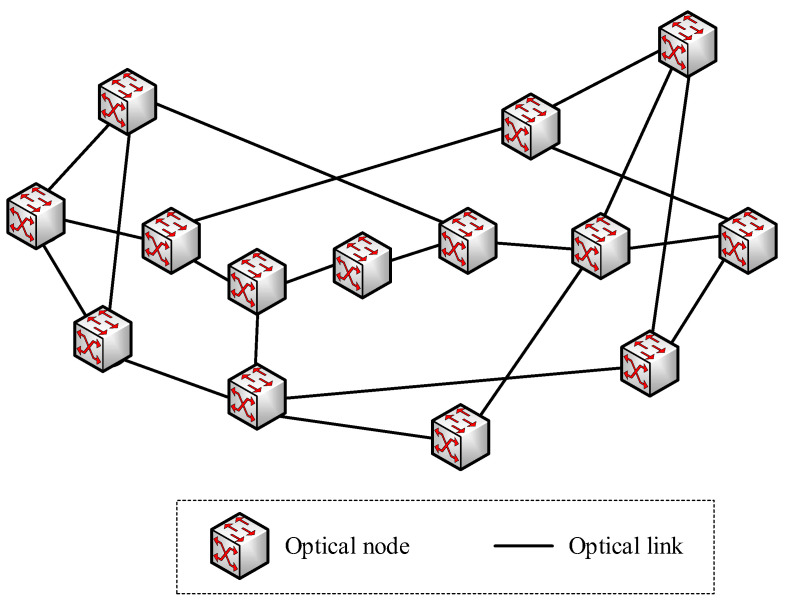
The NSFNET topology used in the numerical simulation.

In order to evaluate the performance of the proposed KA-SVNP algorithm in a high-load environment, numerical simulations are carried out using the 14-node NSFNET topology, as shown in Figure 6. The secure virtual network requests arrive following a Poisson process. The value of K is set to 5 in the K-shortest-path approach. The number of virtual nodes for a secure virtual network request is randomly selected from the interval [2,3].

**Figure 7:**
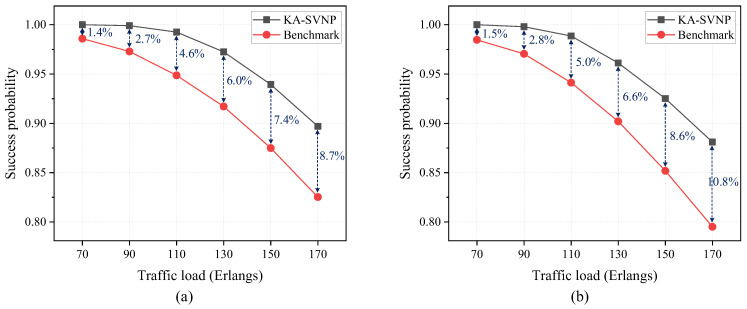
Success probability of secure virtual network requests versus traffic load for the proposed KA-SVNP algorithm and the benchmark algorithm in two scenarios: (**a**) without bandwidth limitation; (**b**) with bandwidth limitation.

Figure 7 presents a comparative analysis of the proposed KA-SVNP algorithm and the benchmark algorithm in terms of the success probability of secure virtual network requests, where the scenarios without bandwidth limitation and with bandwidth limitation are considered in Figure 7a,b, respectively. The benchmark algorithm is the traditional virtual network provisioning algorithm with stochastic available path selection, which does not take into account the key adaption. The key requirement for each secure virtual network request is randomly selected from the interval [1,4] kbps, while the information-theoretically secure key resources (i.e., key rates) for each substrate link are 50 kbps. In Figure 7a, the number of bandwidth resources is sufficient. In Figure 7b, each substrate link provides 40 wavelength channels (each at 50 GHz) of bandwidth resources, while each secure virtual network request requires one wavelength channel at 50 GHz. From Figure 7 we can see that the proposed KA-SVNP algorithm demonstrably outperforms the benchmark algorithm in both scenarios. For example, when the traffic load is 170 Erlangs, the improvement in the success probability of secure virtual network requests for the KA-SVNP algorithm relative to the benchmark algorithm can reach 8.7% and 10.8% in the scenarios without bandwidth limitation and with bandwidth limitation, respectively, thereby demonstrating the efficiency of the proposed KA-SVNP algorithm. Hence, the key supply (i.e., the information-theoretically secure key resources for each substrate link) and key demand (i.e., the key requirement of the secure virtual network request) can be adaptive by the selection of the physical path with the maximum value of $D_{p(e_{r})}$ in the proposed KA-SVNP algorithm.

**Figure 8:**
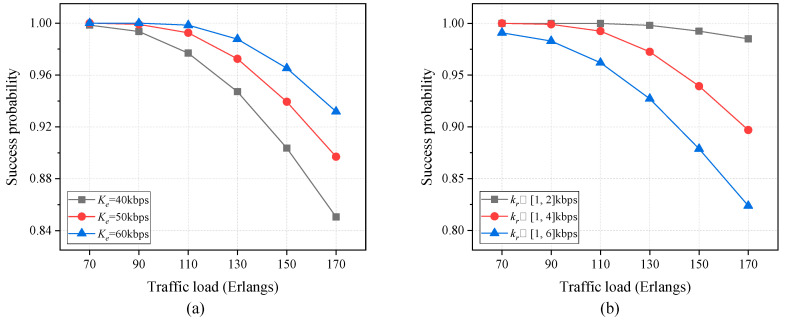
Success probability of secure virtual network requests versus traffic load for different PLS-based (**a**) key resources and (**b**) key requirements.

The results of the success probability of secure virtual network requests as a function of traffic load are plotted in Figure 8, where the scenario without bandwidth limitation is considered. In Figure 8a, the comparative analysis of different information-theoretically secure key resources is presented, where the key requirement for each secure virtual network request is randomly selected from the interval [1,4] kbps. In addition, Figure 8b illustrates the comparison of different key requirements, with the PLS-based key rate for each substrate link set at 50 kbps. It can be seen that the success probability of secure virtual network requests declines progressively as the traffic load rises. This is due to either an increase in the arrival rate or a decrease in the departure rate. As shown in Figure 8a, an increase in the PLS-based key rate results in a corresponding rise in the success probability of secure virtual network requests, due to the expansion of the total available key resources. As illustrated in Figure 8b, the success probability of secure virtual network requests declines in conjunction with an increase in key requirements. This is due to an increase in the total key requirements. Consequently, in order to accommodate a larger number of secure virtual network requests, it may be necessary to either increase the value of $K_{e}$ or reduce the range of $k_{r}$. However, this may result in a trade-off between cost and flexibility.

## 5. Conclusions

This paper presents an investigation of the secure virtual network provisioning over KPONs. The information-theoretically secure keys are generated via the PLS-based method to ensure the information-theoretic security of data transfer within virtual networks. A layered architecture for enabling efficient configuration of secure virtual networks over the KPON is illustrated. Furthermore, the network model is formulated and the key adaption metric is defined. A KA-SVNP algorithm is designed relying on the dynamic adaptation of the key supply and key demand, where the physical path for each virtual link of the secure virtual network request is specifically selected based on the value of $D_{p(e_{r})}$. The emulation testbed is established, and the emulation results for the latency are analyzed. In particular, the millisecond latency for the establishment/deletion of a secure virtual network over the KPON can be achieved. Furthermore, numerical simulations are performed, and the simulation results for the success probability of secure virtual network requests versus the traffic load are analyzed. With regard to the success probability of secure virtual network requests, the proposed KA-SVNP algorithm is observed to outperform the benchmark algorithm by 8.7% under a traffic load of 170 Erlangs. Moreover, in order to increase the success probability of secure virtual network requests, it is possible to either increase the value of $K_{e}$ or reduce the range of $k_{r}$. In future work, we will further conduct simulation experiments on large-scale carrier-grade topologies to verify the generalization ability of the proposed algorithm in real network scenarios. Furthermore, machine learning methods are expected to be utilized to enhance the performance of secure virtual network provisioning.

## Data Availability

The original contributions presented in this study are included in the article. Further inquiries can be directed to the corresponding author.
